# Cytokines and Chemokines in HBV Infection

**DOI:** 10.3389/fmolb.2021.805625

**Published:** 2021-12-02

**Authors:** Shihong Zhong, Tianling Zhang, Libo Tang, Yongyin Li

**Affiliations:** State Key Laboratory of Organ Failure Research, Guangdong Provincial Key Laboratory of Viral Hepatitis Research, Department of Infectious Diseases, Nanfang Hospital, Southern Medical University, Guangzhou, China

**Keywords:** hepatitis B virus, cytokine, chemokine, immune response, liver disease

## Abstract

Chronic hepatitis B virus (HBV) infection remains a leading cause of hepatic inflammation and damage. The pathogenesis of chronic hepatitis B (CHB) infection is predominantly mediated by persistent intrahepatic immunopathology. With the characterization of unique anatomical and immunological structure, the liver is also deemed an immunological organ, which gives rise to massive cytokines and chemokines under pathogenesis conditions, having significant implications for the progression of HBV infection. The intrahepatic innate immune system is responsible for the formidable source of cytokines and chemokines, with the latter also derived from hepatic parenchymal cells. In addition, systemic cytokines and chemokines are disturbed along with the disease course. Since HBV is a stealth virus, persistent exposure to HBV-related antigens confers to immune exhaustion, whereby regulatory cells are recruited by intrahepatic chemokines and cytokines, including interleukin-10 and transforming growth factor β, are involved in such series of causal events. Although the considerable value of two types of available approved treatment, interferons and nucleos(t)ide analogues, effectively suppress HBV replication, neither of them is sufficient for optimal restoration of the immunological attrition state to win the battle of the functional or virological cure of CHB infection. Notably, cytokines and chemokines play a crucial role in regulating the immune response. They exert effects by directly acting on HBV or indirectly manipulating target immune cells. As such, specific cytokines and chemokines, with a potential possibility to serve as novel immunological interventions, combined with those that target the virus itself, seem to be promising prospects in curative CHB infection. Here, we systematically review the recent literature that elucidates cytokine and chemokine-mediated pathogenesis and immune exhaustion of HBV infection and their dynamics triggered by current mainstream anti-HBV therapy. The predictive value of disease progression or control and the immunotherapies target of specific major cytokines and chemokines in CHB infection will also be delineated.

## Introduction

Hepatitis B virus (HBV), a hepatotropic, non-cytopathic DNA virus, is responsible for most cases of viral hepatitis (Seeger and Mason, 2015). Although with the widespread application of two types of available approved treatment, interferons alpha (IFN-α) and nucleos(t)ide analogues (NUCs), which effectively suppress HBV replication, neither of them eliminates the virus mechanistically, leaving the risk of hepatocellular carcinoma (HCC) remains, and being a major cause of morbidity and mortality worldwide.

The clearance of HBV mainly depends on the antiviral effect of the immune system. Over 90% of people infected in adulthood will resolve the infection, presenting as the clinical manifestations of acute self-limiting infection, reflecting an optimal host immune response involving early control of viral replication (Terrault et al., 2018). Serving as the first defense line against HBV insulting, the innate immune arm reacts promptly and mightily. Subsequently, it triggers the formidable adaptive immune response by either killing virus-infected hepatocytes directly or exerting non-cytolytic mechanisms mediated by soluble cytokines (Kapoor and Kottilil, 2014). Therefore, it is safe to deduce that our fully-developed immune system is strong enough to achieve HBV containment. By contrast, unfortunately, over 90% of individuals infected in infancy will progress from acute hepatitis to lifelong chronic infection (Kwon and Lee, 2011). The causes of HBV infection chronicity remain unclear. Infectiousness at an early age is the main element accounting for persistency, which may be partially attributed to the distinct features of innate and adaptive immunity from fetal life to adulthood. Besides, viral load, genotype, route of infection, age, and genetics of the infected host jointly modify the natural course of HBV infection (Fanning et al., 2019). Chronic HBV infection causes liver injury, which is predominantly mediated by persistent intrahepatic immunopathology. The events of repetitively active viral replication, hepatic necroinflammatory, and inflammatory-induced damage repair take place iteratively, which ultimately build the pathological basis of HBV-related liver cirrhosis (LC) and HCC (Liang, 2009).

Although been controversial (Wang S. et al., 2013; Zhang Z. et al., 2020), HBV is deemed to be a stealth virus that escapes surveillance without detected by pattern recognition receptors in infected hepatocytes or acts as a suppressor of the innate defense system by multiple pathways (Yu et al., 2017). With persistent HBV-related antigen (Ag) stimulation, chronic HBV infection puts the immune system into a dilemma where expeditious and appropriate adaptive immune response cannot be evoked or be in an attrition state, leading to the anergy of anti-HBV specific immune response (Fisicaro et al., 2020). In the meantime, cytokines and chemokines, as the essential components of the immune system, undergo tremendous disturbances and participate in the exhaustion of the anti-HBV immune response. Moreover, based on its location and anatomic features, the liver harbors a specific feature of tolerance to pathogens and antigens draining from the gut, thereby avoiding severe immune-mediated damage (Robinson et al., 2016), this, however, renders the stealth virus more rampant in the liver, making it more intractable for the aggrieved adaptive response to tackle. Although the current anti-HBV therapy in clinical practice can significantly inhibit viral replication, there is still a distant way to achieve the current treatment goal of CHB patients. This functional cure is defined as undetectable HBV DNA and hepatitis B surface antigen (HBsAg) loss over a limited treatment period (Terrault et al., 2018). Meanwhile, the treatment response predictors and withdrawal criteria after long-term maintenance of NUCs therapy are still unclear. Therefore, it is urgent to seek predictive indicators of treatment response and new treatment strategies. At present, with the fabulous development of scientific-technical advances, accumulating evidence indicates that the involvement of cytokines and chemokines in the pathogenesis of HBV is more and more unambiguous, providing a proof of concept for immune-modulating therapy. Therapeutic approaches that target cytokines and chemokines associated with the severity of chronic HBV infection sound promising.

In this review, we will systematically discuss the recent literature that elucidates cytokine and chemokine-mediated pathogenesis and immune exhaustion of HBV infection and their dynamics triggered by current mainstream anti-HBV therapies. We also emphasize the potential predictive value of disease progression or control and the immunotherapies targeting specific major cytokines and chemokines in CHB infection.

## Cytokines in Hepatitis B Virus Infection

### Interleukin-1

The inflammatory cytokines Interleukin (IL)-1, including IL-1α and IL-1β, was first identified in the 1940s as an endogenous pyrogen (Menkin, 1943; Dinarello, 1991). IL-1α and IL-1β have similar biological activities (Netea et al., 2010), but the role of IL-1β in HBV has been attached more attention. In chronic HBV infection, HBV with its component, hepatitis B e antigen (HBeAg), have been reported to manipulate a variety of strategies to inhibit both the production and effects of IL-1β. HBeAg significantly inhibits the LPS-induced NLRP3 inflammasome activation and IL-1β production, and also attenuates its downstream pathway of NF-κB activation (Wang et al., 2019). Thereby, these may favor the establishment and maintenance of persistent infection. In contrast, hepatitis B c antigen (HBcAg) promotes LPS-induced NLRP3 inflammasome activation and IL-1β production (Wang et al., 2019). At last, the counteraction turns out to be the upregulation of IL-1β found in the serum, peripheral blood mononuclear cells (PBMCs) and *in vitro* culture primary human hepatocytes (Tian et al., 2019; Zhang Z. et al., 2020; Li and Jiang, 2021). Besides, IL-1β is also linked to IFN-α treatment response. CHB patients responded to IFN-α with clearance of HBeAg and sustained inhibition of HBV replication were accompanied by substantial rises in IL-1β in serum and spontaneous *in vitro* production from PBMC (Lei et al., 2019), suggesting the treatment response predictor and potential therapeutic roles of IL-1β. In addition, the polymorphisms of the genes encoding IL-1β are associated with disease severity in HBV infection and HBV-related hepatic complications, which might serve as a potential genetic biomarker (Tuncbilek, 2014; Dhifallah et al., 2021).

### Interleukin-2

IL-2 was first identified as a T cell growth factor produced primarily by CD4^+^ T cells (Morgan et al., 1976). IL-2 interacts with the intermediate-affinity and high-affinity IL-2 receptors functionally expressed by resting natural killer (NK) cells, CD8^+^ T cells, and lymphocytes following their activation, respectively (Letourneau et al., 2010). IL-2 can boost the proliferation of T cells, the cytolytic activity of NK cells. On the other hand, IL-2 exerts its immunoregulatory effects by promoting the development and suppressive activity of T regulatory (Treg) cells (Liao et al., 2013). Research on the role of IL-2 in HBV infection is dominantly referred to its representative significance for the evaluation of functions in T cells, especially in the HBV-specific T cells during the natural course of HBV infection, as well as its dynamics in response to the anti-HBV treatment (Chokshi et al., 2014; Tan et al., 2018). The levels of IL-2 increase significantly in a time-dependent manner in patients with adefovir dipivoxil or telbivudine treatment, which is contrary to that with entecavir treatment (Piao et al., 2012; Li C. et al., 2013; Gao et al., 2021). Elevated levels of IL-2 on-treatment are associated with HBeAg seroconversion after treatment withdrawal (Chokshi et al., 2014), indicating that IL-2 may serve as an off-treatment predictor. During chronic HBV infection, CD4^+^ T cell exhaustion has the absence of IL-2, which is partially restored by anti-inhibitor molecular treatment (Dong et al., 2019). Additionally, nearly a decade ago, scientists had embarked on a concerted journey to the clinical application of IL-2 but achieved highly heterogeneous results. Some studies showed that recombinant (r) IL-2 acted as an immunomodulatory agent enhancing host immune activity and might benefit CHB patients (Onji et al., 1987). In contrast, others evidenced that IL-2 therapy over short periods did not result in complete clearance of HBV, and treatment with IFN-α alone was preferable to a regimen of IFN-α/IL-2 applied (Nishioka et al., 1987; Bruch et al., 1993). Overall, although the combination of IL-2 and the conventional therapies might be a promising strategy to cure HBV, the suitable concentration, tissue-targeting, and add-on manner remain further clarified.

### Interleukin-4

IL-4 is distinguished as a T helper (Th)2 cytokine that tilts the adaptive response toward humoral immunity by motivating proliferation, differentiation, and antibodies production of B cells, promoting Th2 cells differentiation from naïve CD4^+^ T cells and inhibiting Th1 responses as well as IFN-γ production (Nelms et al., 1999).

During chronic HBV infection, IL-4 is downregulated, compared with those in healthy control (HC), and has an inverse correlation with virus load and HBsAg titers (Gu et al., 2019). Moreover, the expression levels of IFN-γ are gradually elevated, and the expression levels of IL-4 are progressively lowered from the immune tolerance phase to the inactive carrier phase (Li M. et al., 2016), indicating a shift from Th2 to Th1 responses. Interestingly, it has been reviewed that there is a consistent boost of IL-4 after NUCs treatment regardless of the specific agent (Li et al., 2017; Tavakolpour et al., 2017), whereas levels of serum IL-4 are decreased during the treatment with IFNα-2a therapy in virological responders (Park et al., 2012). As a competent cytokine, IL-4 can suppress the expression and the replication of HBV in different HCC lines (Lin et al., 2003; Yao et al., 2011). In addition, it has been suggested that the IL-4 (-590) CT genotype is a vital protective factor for the development of hepatitis among chronic HBV carriers. In contrast, the genetic variants in IL-4 -590C/T and -33C/T polymorphisms may be a risk factor for CHB in Chinese males but not for HBV-related LC or HCC (Lu et al., 2014; Saxena et al., 2014).

### Interleukin-6

IL-6 a cytokine mainly produced by activated monocytes in response to inflammatory stimuli, is involved in a wide range of pleiotropic actions that affect the functions of a variety of lymphoid cells (Van Snick, 1990). IL-6 signals through membrane-bound and soluble IL-6 receptor (sIL-6R), mediating a classic signaling pathway or a trans-signaling pathway, respectively. Of note, the regenerative or anti-inflammatory activities of IL-6 are mediated by traditional signaling, whereas pro-inflammatory responses of IL-6 are mediated by trans-signaling (Rose-John, 2012).

The roles of IL-6 in HBV infection, including acute hepatitis B (AHB), CHB, and HBV-related diseases, have been widely described. However, the interpretation of the role of IL-6 in HBV is complicated because the sIL-6R can mediate trans-signaling and is implicated in a series of inflammatory diseases. During HBV infection, IL-6 is found markedly higher mediated by various pathways and closely correlated to the degree of hepatocyte damage in the HBV-related disease spectrum. Specifically, Li et al. have evidenced that HBV middle S protein and HBcAg enhance IL-6 production via p38, ERK, and NF-κB pathways (Li Y.-X. et al., 2016; Chen et al., 2017) while HBx protein not only promotes complement component 3 production by inducing IL-6 secretion from hepatocytes in mice, but stimulates the production of IL-6 in a MyD88-dependent manner, leading to HBV-mediated liver carcinogenesis (Xiang et al., 2011; Yuan et al., 2016). Furthermore, a sustained high level or dynamic elevated level of serum IL-6 indicates higher mortality in patients with HBV-acute-on-chronic liver failure (ACLF) (Zhou et al., 2020). Another research reported that HBcAg established a proinflammatory microenvironment by promoting the production of IL-6 of M2 macrophages via the TLR2 pathway (Yi et al., 2020). As for the dynamics of IL-6 upon anti-HBV treatment, two studies showed that Pegylated (Peg)-IFNα therapy induced a distinct and rapid up-regulation of IFN signaling pathway that coincided with increased detection of IL-6 (Tan et al., 2014), and IL-6 levels at 3rd and 6th months after treatment showed a predictive value of sustained virological response (Park et al., 2012). Paradoxically, another study has evidenced that, compared with baseline, the Peg-IFNα group showed a significant decrease in IL-6 during 3–6 months of treatment (Li MH. et al., 2021). In addition, serum levels of IL-6 do not reflect the inflammatory activity of hepatitis and have no predictive value of positive response to the IFN-α therapy in children with CHB (Gora-Gebka et al., 2003). In contrast to the controversy over IFN-α therapy, patients with NUCs treatment show a consistent decrease in the levels of IL-6 (Lu et al., 2008; Yu et al., 2021).

As a pleiotropic cytokine, IL-6 exerts inhibiting effects on HBV through multiple layer mechanisms. IL-6 inhibits HBV entry and transcription through sodium taurocholate cotransporting polypeptide (NTCP) down-regulation, targeting the epigenetic control of the nuclear covalently closed circular (ccc)DNA minichromosome, and increasing the enhancer activity of HBV enhancer 1 through signal transduction pathways (Palumbo et al., 2015). Nevertheless, the pernicious side of IL-6 on HBV infection stands out. Several studies have revealed that HBV exploited the IL-6 signal pathway to manipulate the development of LC and HCC (Chang et al., 2015; Kao et al., 2015; Zhou et al., 2018). HBV-induced mitochondrial reactive oxygen species production leads to the sustained activation of the IL-6-STAT3 pathway and ultimately contributes to HCC (Yuan et al., 2016). Overall, as there are beneficial and detrimental effects of IL-6 in HBV infection, more mechanistic research is needed to interpret this double-edged sword judiciously.

### Interleukin-12

IL-12 is primarily produced by dendritic cells (DCs), monocytes, and macrophages, and to a lesser extent by B cells, whose role is predominantly associated with the differentiation of naïve T cells into Th1 cells, serving as a linkage between innate to cellular immunity (Heufler et al., 1996). It also promotes the **e**xpansion and survival of activated T cells and NK cells and modulates the cytotoxic activity of cytotoxic T lymphocytes (CTLs) and NK cells (Rossol et al., 1997). During the adaptive immune response, IL-12 primes Ag-specific T-cells for high IFN-γ production. IL-12 can also act as an adjuvant for humoral immunity by enhancing antibody (Ab) production by B cells (Metzger et al., 1997).

HBV-induced IL-12 expression involves activating the PI3K-Akt pathway by HBx, leading to the transactivation of the IL-12 p35 and p40 promoters (Wang et al., 2015). Serum levels of IL-12 are associated with alanine aminotransferase (ALT) levels, and the highest serum levels of IL-12 was accompanied by HBeAg or HBsAg seroconversion in both AHB and CHB patients (He et al., 2012; Wu et al., 2015), suggesting serum levels of IL-12 may be an available marker to evaluate cellular immunity for HBV infection. Elevated IL-12 rescues the anti-viral function of exhausted HBV-specific CD8^+^ T cells, enhances the anti-virus properties of cytotoxicity, polyfunctionality, and multispecificity of HBV-specific T cells. Furthermore, IL-12 significantly decreases the pro-apoptotic molecule Bim, which can mediate premature attrition of HBV-specific CD8^+^ T cells (Xiong et al., 2007; Wu et al., 2015). Co-stimulation with IL-12 is found to significantly augment the HBs/e/cAg-specific secretion of IFN-γ (Vingerhoets et al., 1998; Szkaradkiewicz et al., 2005). Besides, there is a clear consensus that the CHB patients with current available anti-HBV treatment presented higher amounts of IL-12 are associated with favorable outcomes (Ozkan et al., 2010; Yang et al., 2020).

As a promising therapeutic cytokine, IL-12 has been well explored in human studies and murine experimental models. IL-12-based vaccination therapy restores systemic HBV-specific CD4^+^ T cell responses, elicits robust intrahepatic HBV-specific CD8^+^ T cell responses, and facilitates the generation of HBsAg-specific humoral immunity in a mouse model of HBV carrier (Zeng et al., 2013). Moreover, combined with a plasmid expressing IL-12, HBV DNA vaccine has a strong antigenicity in humoral and cellular immunities, enhances T cell reactivity to HBV and IFN-γ production, and showed 50% of virological response rate in CHB carriers under lamivudine treatment (Du et al., 2003; Rigopoulou et al., 2005; Yang et al., 2006). Additionally, treatment with IL-12 at suitable doses is safe and tolerable, and appears to be active against HBV in CHB patients (Carreno et al., 2000). Nevertheless, controversy still exists as other studies point out that IL-12 induces a strong immunosuppressive reaction in the liver of chronic woodchuck hepatitis virus carriers that counteracts the antiviral effect of the treatment, and the antiviral activity of rIL-12 does not appear to be advantageous in comparison to other currently available therapies in CHB patients (Zeuzem and Carreno, 2001; Otano et al., 2012). Therefore, more intensive research is needed before the application of IL-12 into clinical practice.

### Interleukin-21

IL-21 is another pleiotropic cytokine, which is dominantly derived from follicular T helper (Tfh) cells, Th17 cells, and activated NKT cells (Zeng et al., 2007). Its receptor is widely expressed on various immune cells, endowing it a multifunctional role in the pathogenesis and prognosis of HBV infection.

In the course of natural HBV infection, similar to other proinflammatory cytokines, IL-21 elevates significantly at hepatic flare in AHB, CHB, and HBV-ACLF, especially in those achieving resolve or HBsAg seroconversion subsequently (Chen et al., 2014; Yoshio et al., 2018; Du et al., 2021). IL-21 has a pivotal implication in the instruction of antiviral treatment. Serum levels of IL-21 at treatment week 12 independently predicted HBeAg seroconversion in the first year of treatment in CHB patients with telbivudine treatment or salvage therapy (Ma et al., 2012; Li et al., 2021b). Additionally, serum IL-21 levels at 0, 12, 24, 52, and 104 weeks after discontinuance of entecavir are significantly higher in the durable virological remission group than in the virological relapse group (Huang et al., 2021).

Numerous studies have provided mechanistic evidence for the role of IL-21 in HBV infection. IL-21 promotes the proliferative capacity of HBV-specific CD8^+^ T cells, down-regulates expression of the inhibitory receptors PD-1 and TIM-3, and further boosts and sustains the antiviral effects of HBV-specific CD8^+^ T cells by enhancing both cytolytic and noncytolytic pathways (Li et al., 2015; Shen et al., 2021), thereby, contributing to viral containment and clearance. In addition, IL-21 also reduces HBV replication by inhibiting IL-10 secretion (Li et al., 2015). Our group has found that circulating CXCR5^+^CD4^+^ T cells favor HBeAg seroconversion through IL-21 in patients with CHB infection (Li Y. et al., 2013), and IL-21R deficient impairs the production of HBV-specific IFN-γ from intrahepatic CXCR5^+^CD8^+^ T cells (Li et al., 2020). Moreover, IL-21 is reported to take part in determining the age-dependent outcome of HBV infection. In an HBV mouse model, adult mice elicited a robust, diverse, long-lived HBV-specific T cell response and had increased numbers of IgG-expressing B cells in an IL-21 dependent manner, resulting in HBV clearance. Conversely, young mice and the absence of the IL-21R in adult mice resulted in Ag persistence (Publicover et al., 2011). Interestingly, IL-21 is also involved in the vertical transmission of HBV. Impaired generation of serum IL-21, Tfh cell, and plasma B cell are associated with vertical transmission of HBV to newborns, which can be improved by HBV vaccine booster-induced IL-21 production in an HBV mouse model (Vyas et al., 2019; Wang et al., 2021). Notably, the excessive or persistent inflammatory response of IL-21 leads to liver damage, HBV-related LC, and HCC (Liu B. et al., 2017; Wu et al., 2021). Taken together, a successful treatment strategy of IL-21 should be personalized tailor-made.

### Th17-Associated Cytokines

Th17 cells are a subset of T helper cells that play a critical role in host defense against pathogens insult. Th17 cells arise from naïve Th0 cells initiated by IL-1, transforming growth factor-β1 (TGF-β1) and IL-6, combined with the activation of transcription factor retinoic acid receptor-related orphan nuclear receptor gamma t, driving the development of various inflammation-related as well as autoimmune diseases (Dong, 2008). IL-17 and IL-22 are the main function executors of Th17 cells (Qu et al., 2013). Generation of the pathogenic Th17 cells requires IL-23 stimulation, and IL-23 amplifies the Th17 cell responses and causes inflammation injury (McKenzie et al., 2006). In CHB patients, Th17 cells are significantly elevated. They initiate immune-mediated pathogenesis and have a critical role in the process of HBV-related LC whose underlying mechanisms are greatly attributed to Th17-secreted cytokines.

### Interleukin-17

IL-17 is an essential proinflammatory cytokine family that consists of six family members (IL-17A to IL-17F) encoded by separate genes, of which IL-17A is the representative effector cytokine secreted by Th17 (Jin and Dong, 2013). Expression of the IL-17R has been detected on almost all liver cell types (Ge and You, 2008), leading to the extensive and intricate pathogenesis process in the liver tissue during HBV infection. Similar to other proinflammatory cytokines mentioned above, peripheral and intrahepatic IL-17 was significantly increased in the setting of HBV infection, and correlated positively with the severity of liver damage and LC and HCC (Bao et al., 2017).

IL-17A suppresses HBV replication in a noncytopathic manner and over-expression of the antiviral proteins myxovirus resistance A and oligoadenylate synthetase mRNA (Wang B. et al., 2013). However, inappropriate, excessive, and non-specific Th17 effector responses are involved in the pathogenesis of liver damage, even liver failure. It has been described that the Th17-IL-17 axis is simultaneously the fuel and the flame of a sustained proinflammatory and profibrotic environment during CHB infection through a stepwise manner (Paquissi, 2017). During HBV infection, HBxAg-activated hepatic stellate cells (HSCs) recruit more Th17 cells into the liver that could, in turn, stimulate the proliferation and fibrotic marker secretion of the HSCs mediated by IL-17A and IL-22, forming a positive feedback loop that aggravates the progression of chronic liver disease with HBV infection (Tan et al., 2013; Zhang H. et al., 2020). In addition, counteraction between Th17 and Treg cell has been attached much attention by scientists (Su et al., 2013). The expression of Treg and Th17 cells are both increased, but the ratio of Treg/Th17 is significantly decreased in patients with HBV infection (Feng et al., 2015). Reduced ratio of Treg cells and IL-10, TGF-β1 levels to Th17 cells and IL-17 levels correlate with HBV DNA suppression in CHB patients undergoing entecavir treatment, especially in the treatment response group (Zhang et al., 2010; Yu et al., 2013). In contrast, imbalance of Treg and Th17 cells might play an essential role in the occurrence, development, and outcome of CHB, although distinct pathways await elucidation (Su et al., 2013).

### Interleukin-22

IL-22 is a member of the IL-10 family produced primarily by Th17 and NK cells (McAleer and Kolls, 2014). IL-22 is characterized by dual effects in the context of inflammation, and this has been attributed to its coexpression along with IL-17 (McAleer and Kolls, 2014). Evidence showed that IL-22 served as an effective adjuvant to enhance cellular immune responses during HBsAg DNA vaccination by inducing Tc17 cells to break tolerance in HBsAg transgenic mice (Wu et al., 2013). On the other hand, serum IL-22 levels and liver-infiltrating IL-22^+^ cells increased stepwise from CHB to atypical hyperplasia and HCC, suggesting poor prognostic indicators for HCC (Shi et al., 2020). Additionally, IL-22 exacerbated chronic liver inflammation and fibrosis by CXCL10-and CCL20-recruited Th17 cells in HBV-infected patients and HBV Tg mice (Zhao et al., 2014). Collectively, the interpretation of the role of IL-22 in HBV infection requires prudent and comprehensive consideration for the specific disease status and distinct intrahepatic microenvironments.

### Interleukin-23

IL-23, a member of the IL-12 family of cytokines with proinflammatory properties, is expressed principally by the macrophages and DCs, with the main biological functions of stimulation of DC antigen presentation, generation, and maintenance of Th17 cells (McKenzie et al., 2006). HBV induces IL-23 production in antigen-presenting cells and causes liver damage via the IL-23-IL-17 axis. HBsAg efficiently induces IL-23 secretion by myeloid DCs in a mannose receptor-dependent endocytosis manner while HBcAg-stimulated IL-23 secretion is mannose receptor- and endocytosis-independent (Wang Q. et al., 2013). Both IL-23 and IL-23R are remarkably elevated in biopsied liver tissues in HBV infection patients. Elevated IL-23 amplifies Th17 cell responses and liver inflammation. Furthermore, IL-23 upregulates IL-23R expressions on macrophages, enhancing macrophage-mediated angiogenesis (Wang Q. et al., 2013). Persistent IL-23 generation by liver inflammatory macrophages responding to damaged hepatocytes after chronic HBV infection alters macrophage function and shapes a pro-cancer milieu for HCC (Zang et al., 2018). Overall, these data provide mechanistic insights into the therapeutic potential of IL-23.

### Interferon

The IFN family stands on the first line of defense activated upon viral infection and is paramount for controlling viral replication and dissemination. Although initially named after their function in interfering with viral replication, IFNs have more functional roles since being discovered for more than half a century.

IFNs bind IFN receptors on the surface of neighboring and/or immune cells, triggering a signaling cascade to induce a suite of IFN-stimulated genes (ISGs) that directly mediate the antipathogenic effects of IFNs (Schoggins et al., 2011). There are three distinct IFN families (IFN-I, -II, and -III), among which the well-characterized IFNs, IFN-α and IFN-β belong to the IFN-I family (McNab et al., 2015), and IFN-γ is the single gene product of the IFN-II family (Fenimore and Young, 2016).

Currently, IFNs are used therapeutically, with the most noteworthy example being the treatment of HBV and HCV infection, and this clinical practice has demonstrated the extraordinary value of IFNs. The fundamental mechanisms that control HBV infection with IFN-α treatment are relatively well recognized. IFN-α exerts both direct antiviral, which have been well elucidated (Sadler and Williams, 2008), and host immunomodulation effects (Biron, 2001). Specifically, IFN-α inhibits the HBV transcription and replication cycle by transcription and epigenetic modification pathways observed in human and mouse models (McNab et al., 2015). As refer to the immunoregulatory functions, numerous studies have suggested that IFN-α augments IL-27-dependent IFN-stimulated gene, induces spontaneous production of tumor necrosis factor-α (TNF-α), IL-1β (Ren et al., 2018) and improves IL-2 activity (Saxena et al., 1985), as well as triggers NK cell functionality and HBV-specific T cell responses (Bruder Costa et al., 2016). Of note, Tian et al. emphasized that effects of IFN-I on HBV replication were determined by viral load. IFN-I suppresses HBV replication when viral load is high and enhances HBV replication when viral load is low via transcriptional and post-transcriptional regulations (Tian et al., 2011). However, some patients suffer from IFN-α treatment resistance by inducing CD24^+^CD38^hi^ B cell and IFN-α/γ-STAT1-PD-L1 axis-mediated downregulating functions of T cells and NK cells (Fu et al., 2020; Liu et al., 2020) as well as producing anti-IFN-α Abs (Porres et al., 1989).

HBV-specific T cells execute function partially depending on IFN-γ. IFN-γ inhibits HBV replication and reduces cccDNA in hepatocytes by inducing deamination and cccDNA decay (Xia et al., 2016). HBV-specific IFN-γ producing CD4^+^ T cells are associated with viral clearance (Wang et al., 2020). In addition, IFN-III also participates in HBV clearance. IFN-III-induced IL-10 plays a vital role in producing HBV restriction factor CBFβ (Xu et al., 2019), and nucleotide analogues show an additional pharmacological effect by inducing IFN-λ3 production, which further induces ISGs and results in a reduction of HBsAg production (Murata et al., 2018).

### Tumor Necrosis Factor-Alpha

TNF-α is a potent proinflammatory cytokine mainly produced by monocytes and macrophages (Wajant et al., 2003). It elicits a particularly broad spectrum of cell proliferation, differentiation, and apoptosis in response to inflammation, infection, injury, and other environmental challenges (Baud and Karin, 2001). TNF-α plays a dichotomous role in HBV infection, which acts as a mediator of anti-HBV immunity and induces liver inflammation, and sustained liver inflammation leads to liver fibrosis. It correlates with ongoing inflammation among chronic HBV patients with LC, which is likely attributed to the expression of biosignatures of apoptosis and activation in immune cells (Wang et al., 2020). It is reported that TNF-α producing cells are the dominant population among HBV-specific CD4^+^ T cells and are associated with liver damage, but not viral clearance (Barathan et al., 2021). Of note, with the clinical application of anti-TNF-α therapy in inflammatory bowel and rheumatic diseases, CHB patients are faced with a challenge of reactivation of hepatitis (Lee YH. et al., 2013). Therefore, anti-HBc-positive patients undergoing anti-TNF therapy need to be carefully monitored, and prophylactic antiviral treatment is usually of great significance (Schwabe and Brenner, 2006).

## Anti-Inflammatory Cytokines in Hepatitis B Virus Infection

### Interleukin-10

IL-10 is a paramount regulatory cytokine that executes most, if not all, of the anti-inflammatory functions of the regulatory immune cells, namely Treg, T follicular regulatory (Tfr), Breg cells, and Myeloid-derived suppressor cells (Redford et al., 2011). It has a central role in infection by limiting the immune response to pathogens and preventing excessive immune activation and damage to the host. It also impedes pathogen clearance and leads to persistent infection. During CHB infection, serum IL-10 is elevated in the active immune group compared with immune tolerant, inactive carrier state, and HC groups. It is positively correlated with ALT and aspartate aminotransferase levels and hepatic flares (Wang et al., 2017). Moreover, increased circulating IL-10^+^ Bregs and Tfr cells are associated with poor virus eradication and liver injury in CHB (Wang et al., 2014). IL-10-producing Breg cells suppress HBV-specific CD4^+^ and CD8^+^ T cell responses but enhance Treg cells in chronic HBV infection (Das et al., 2012). Nevertheless, studies also reveal the favorable characteristics of IL-10 on HBV infection. It was reported that higher serum levels of IL-10 in HBeAg-positive patients were correlated with early, spontaneous HBeAg seroconversion (Wu et al., 2010), and IL-10/HBV DNA ratio was identified as an early positive predictor for response to IFN-α treatment (Yan et al., 2015).

### Interleukin-35

IL-35 is a relatively newly discovered member of the IL-12 cytokine family that has been shown to predominately exert an immunosuppressive effect on T cells (Neurath, 2008). While IL-10 and TGF-β are the most commonly studied immunosuppressive cytokines, the recently identified IL-35 is found to harbor the abilities of not only suppressing effector T cell responses directly (Tao et al., 2018) but also expanding regulatory responses by propagating infectious tolerance and generating a potent population of IL-35-expressing inducible Tregs (Olson et al., 2013). Specifically, IL-35 stimulation elevates viral-specific Tregs, accompanied by increased expression of Foxp3 mRNA and IL-10 production, and decreases IL-17 secretion and STAT3 phosphorylation in CD4^+^ T cells, resulting in an imbalance of viral specific Treg/Th17 cells and thereby contributes to viral persistence (Yang et al., 2019). Additionally, IL-35 dampen non-specific and HBV-specific Th9 cells activity in HBV-related HCC patients (Zhang Q. et al., 2021). Accordingly, IL-35 might be pivotal for developing new therapeutic approaches for hepatitis B.

### Transforming Growth Factor-Beta

TGF-β, another anti-inflammatory cytokine, plays a fundamental role in homeostasis through manipulating cell proliferation, extracellular matrix (ECM) synthesis and degradation, mesenchymal-epithelial interactions during embryogenesis, mediation of cell and tissue responses to injury, control of carcinogenesis, and modulation of immune functions (Verrecchia and Mauviel, 2002). TGF-β exerts dual regulatory functions in the immune system in response to HBV infection. TGF-β stimulates the differentiation of Th17 cells and thus, as mentioned above, favors inflammatory conditions. This contrasts sharply with its anti-inflammatory effects mediated by boosting the activities of Treg cells (Karimi-Googheri et al., 2014). The net benefits from TGF-β are context-dependent which may help to explain the conflicting studies about its role on HBV infection hitherto (Murata et al., 2009). Being recognized as a major profibrogenic cytokine, TGF-β signaling contributes to all stages of liver disease progression from initial liver injury through inflammation and fibrosis to cirrhosis and HCC (Dooley and ten Dijke, 2012). HBV-encoded pX oncoprotein amplifies TGF-β family signaling through direct interaction with Smad4, which serves as a potential mechanism of hepatitis B virus-induced liver fibrosis (Lee et al., 2001). Indeed, functions exerted by TGF-β are not immutable, as it can shift from tumor suppression to oncogenesis accompanied by the tumor development and is adjusted by HBV (Massague, 2008; Giannelli et al., 2014).

### Other Cytokines

In addition to the cytokines reviewed above, there are still many other cytokines that have directive implications in the possibility of designing immunotherapies for CHB patients. IL-7 secreted by inflamed hepatocytes, regulated TCR-mediated activation of human mucosal-associated invariant T cells enriched in the human liver, licensing them to dramatically increase Th1 cytokines and IL-17A production, which may benefit HBV eradication (Tang et al., 2013). A combination between IL-15 and IFN-α induces unprecedented efficacy of functional and specific cellular immunity in HBV transgenic mice (Di Scala et al., 2016). Furthermore, liver over-expression of IL-15 suppresses HBV replication in an IFN-β-dependent manner in mice (Yin et al., 2012). Besides, Jo et al. have evidenced that a combination between IL-12 and IL-18, secreted by monocytes, triggered activation of innate human cells in human liver, resulting the production of a high levels of IFN-γ (Jo et al., 2014). Likewise, IL-18 can inhibit HBV replication in the liver of transgenic mice (Kimura et al., 2002).

## Chemokines in Hepatitis B Virus Infection

Immune cells, as mentioned above, are the predominant source of various cytokines that continuously circulate between the lymph and blood under a homeostasis state. Once encounter pathogens, they take prompt reaction to migrate into the infection site to launch a battle. This is mainly mediated by chemokine ligand and receptor pair, which are critical to achieving precise and efficient immune response and are no exception in HBV infection. Chemokines, also known as chemotactic cytokines, are a family of small (8–12 kDa) cytokines or signaling proteins that exert their functions mainly by inducing directional movement of leukocytes and other cell types, including endothelial and epithelial cells (Raman et al., 2011). These small-molecule ligands are divided into four families based on the positioning of the conserved N-terminal cysteine residues: C, CC, CXC, and CX3C. They modulate biological processes consistently through interactions with seven-transmembrane G protein-coupled receptors (Zlotnik and Yoshie, 2000). The vast majority of known chemokines belong to the CC and CXC families. Of note, although chemokines are best known for their initially identified trafficking and guiding effects, they were later found to orchestrate a variety of additional processes, including the proliferation, differentiation, and activation of cellular responses (Atretkhany et al., 2016). Here, we will elucidate the roles of several fundamental chemokines on HBV infection.

The most critical CXC family chemokine ligand and receptor pairs related to HBV infection are CXCL9/10/11-CXCR3 and CXCL13-CXCR5. Levels of serum CXCL9, CXCL10, CXCL11, CXCL13 are elevated in patients with HBV infection compared with HCs (Kakimi et al., 2001; Keating et al., 2014). Interestingly, the increase of these chemokines happens at hepatitis with the subsequent decline of HBsAg in AHB patients and HBV-inoculated chimpanzees with HBsAg loss, indicating they might be hallmarks of functional cure of AHB or CHB patients (Yoshio et al., 2018). Numerous studies have identified the role of CXCL10, also termed IP-10, in the pathogenesis of HBV. Zhou et al. have demonstrated that HBx upregulates CXCL10 expression in a dose-dependent manner through activation of NF-κB, thereby enhancing the migration of peripheral leukocytes into the liver (Zhou et al., 2010). In an experiment exploiting an HBV transgenic mouse model, it is observed that blocking CXCL9 and CXCL10 reduces the recruitment of Ag-nonspecific lymphocytes as well as ameliorates the severity of liver diseases without affecting the effects of HBV-specific CTL (Kakimi et al., 2001; Sitia et al., 2002). On the other hand, CXCL10 is emphasized as a useful predictive indicator of disease progress and treatment response. Higher baseline serological and histological and slow reduction of CXCL10 levels indicates better prognoses in CHB patients with NUCs or Peg-IFN treatment (Sonneveld et al., 2013; Guo et al., 2016). Besides, pre-treatment CXCL9 level also has the potential to select CHB patients who can respond to Peg-IFN, especially in HBeAg-negative patients with low viral loads (Lee I.-C. et al., 2013). CXCL13, also known as B lymphocyte chemoattractant, is expressed by follicular DCs or stromal cells in lymphoid organs (Ohmatsu et al., 2007). CXCL13 is known for guiding homing of B cells, and subsets of T cells expressed CXCR5 to lymphoid follicles to form secondary lymphoid organs (Stone, 2017), and is critical for the onset and maintenance of humoral immunity. Our team previously focused on the role of CXCL13-CXCR5-mediated HBV-specific immune response. It demonstrated that increased expression of intrahepatic CXCL13 favored the recruitment of CD19^+^ B cells and CXCR5^+^CD8^+^ T cells into the liver to shape a favorable anti-HBV immune milieu (Li et al., 2020). Besides, plasma CXCL13 can serve as a biomarker for GC activity (Havenar-Daughton et al., 2016). Increased plasma CXCL13 is distinctly observed in patients who achieve immune control of CHB infection (Liu C. et al., 2017). CXCL8 (IL-8) and CXCL12 (SDF-1) are two chemokines that have been relatively enthusiastically studied. An environment enriched in IL-7 and IL-15 licenses HBV-specific T cells to secrete CXCL8 (Gehring et al., 2011). Elevated levels of CXCL8 are associated with the severity of liver inflammation/fibrosis and resistance to IFN-α therapy (Yang et al., 2014). Moreover, HBV-induced IL8-CXCR1-TGF-β signaling cascade suppresses antitumor immunity against HCC by enhancing the accumulation of intrahepatic Treg cells and venous metastasis of hepatoma cells (Zhang C. et al., 2021). CXCL12-CXCR4 pathway is involved in recruitment and retention of immune cells in CHB patients with advanced liver fibrosis (Wald et al., 2004). HBx increases endoplasmic reticulum stress-dependent CXCL12 expression and induces HBV-induced immune cell recruitment into liver, with over 50% of liver-infiltrating lymphocytes expressing CXCR4. (Wald et al., 2004; Cho et al., 2014).Chemokines from the CC family also play a significant role in the pathogenesis of HBV infection. The CCL5 expression level in serum increases in CHB patients with aggravated liver injury and significantly decreases in cirrhosis patients with advanced liver fibrosis (Hu et al., 2019). A high expression score of CCL15 is significantly associated with the poor prognosis of HCC patients (Li et al., 2021c). CCL17 and CCL22 are induced by the contact of HBV-transfected cells with monocyte-derived DCs, which may favor the recruitment of Th17 and Tc17 cells to liver tissue in CHB (Zhang K. et al., 2020). A protective effect of CCR5Delta32 in recovery from an HBV infection is observed (Thio et al., 2007). Its ligand, CCL16 also shows an encouraging effect on inhibiting the progression of LC via inactivating HSCs (Zhuo et al., 2020). *In vitro* experiments show that CCL19 enhances the frequencies of Ag-responsive IFN-γ+CD8^+^ T cells from patients by approximately twofold. This is further evidenced by mice overexpressing CCL19 with rapid clearance of intrahepatic HBV, likely through increasing intrahepatic CD8^+^ T cells (Yan et al., 2021). In brief, chemokines from the CC family are critical mediators of HBV infection. Still, since the available studies are discrete and superficial, in-depth research is needed to clarify mechanisms and the possibility of therapeutic application.

## Discussion

**FIGURE 1:**
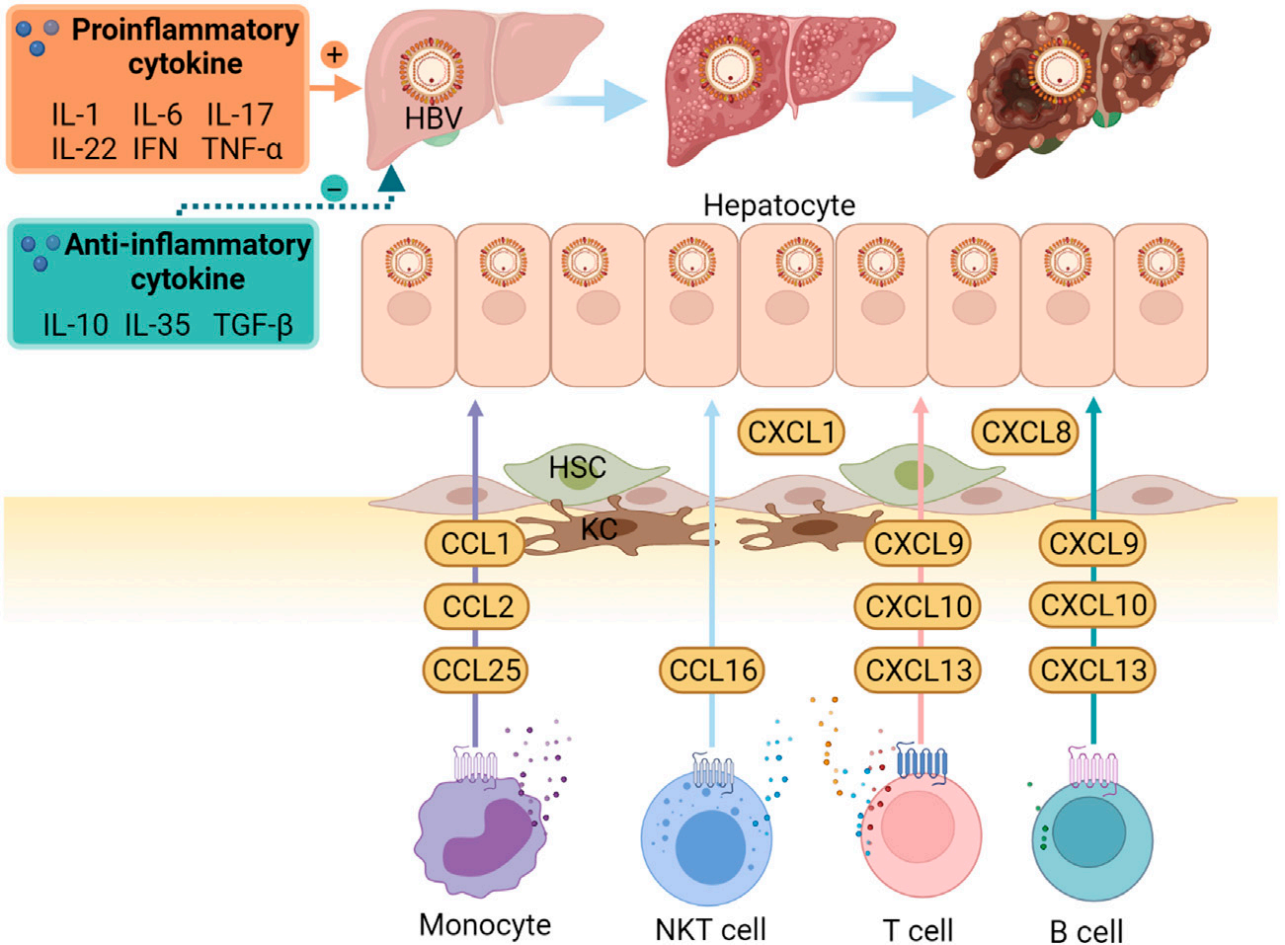
Important cytokines and chemokines in the pathogenesis of HBV infection and HBV-related diseases. When HBV invades, local immune cells respond firstly, especially the KCs. They induce the production of chemokines and cytokines. CCL1, CCl2, and CCL25 contribute to infiltrating monocyte recruitment. CCL16 promotes the migration of NKT cells, whereas others, ligands of CXCR3 and CXCR5, including CXCL9, CXCL10, CXCL13, increase the inflow of T and B cell subsets. In addition, increased intrahepatic CXCL1 and CXCL8 mediate the recruitment of neutrophils. These, together with HSCs changes, result in the disturbance of pro-and anti-inflammatory cytokines, contributing to the development of liver cirrhosis and hepatocellular carcinoma. CCL, C-C Motif Chemokine Ligand; CXCR, C-X-C motif chemokine receptor; HBV, hepatitis B virus; HSC, hepatic stellate cell; KCs, Kupffer cells; NKT, natural killer T. (created with Biorender.com).

Over the past half-century, tremendous progress has been made in understanding the regulation and functions of cytokines and chemokines in the liver. There is no doubt that achieving HBV eradication relies on a well-organized immune response, which is orchestrated considerably by the spatial and temporal expression of cytokines and chemokines. With the assessment of cytokines and chemokines mentioned above, we are delighted to gain a variety of seemingly promising molecules with the predictive value of disease progression or control and immunotherapies target (Figure 1 and Table 1). Actually, we yield far less than expected when transforming them into clinical applications. The measurement of serum or plasma levels of cytokines and/or chemokines is far from established to be used in daily clinical practice for CHB patients. Many chemokines have not been evaluated in-depth, and tailoring the dose of certain cytokine/chemokine administered has crucial implications in optimizing results. In addition, an important block to our understanding of HBV pathogenesis lies in dissecting the critical aspects of the cytokines and chemokines interplay in light of the conditional role these molecules play throughout infection and disease development. It is unreasonable to define the beneficial effects of a single cytokine/chemokine since a few of them play a unique and non-redundant character, and most of them share overlapping and redundant effects. In light of these considerations, defining the fundamental roles of cytokines and chemokines in HBV infection will require the basis of different species, anatomical location, and stages of liver disease development, in combined with the application of more definitive, standard tools as well as strict sample inclusion criteria, which is also meaningful for the design of clinical practice. Therefore, continued research is essential to understand better the complexity of mechanistic pathways and the pleiotropic interactions of cytokines and chemokines. Through the joined hands of scientists from different disciplines, we will eventually be able to win a future without hepatitis B.

**TABLE 1:** Important cytokines and chemokines in HBV infection.

Mediatores	Primary effects	Roles in HBV infection	References
Cytokines			
IL-1	Proinflammatory	Predictor of treatment response to IFN-α	Lei et al., 2019
IL-2	T cell proliferation, NK cell cytolytic activity; promotes Tregs development and suppressive activity	Evaluation of HBV-specific T cell functions; immunomodulatory agent enhancing host immune activity	Chokshi et al. (2014); Tan et al. (2018) Onji et al. (1987)
IL-4	Promotes Th2 cells differentiation and humoral immunity	Suppresses the expression and replication of HBV in different HCC lines; downregulated in CHB patients	Lin et al. (2003); Yao et al. (2011); Gu et al. (2019)
IL-6	Pleiotropic actions that affect the functions of a variety of lymphoid cells	Inhibits HBV entry and transcription; manipulates the development of LC and HCC	Palumbo et al. (2015); Chang et al. (2015); Kao et al. (2015); Zhou et al. (2018)
IL-12	Promotes cellular immunity and modulates the cytotoxic activity of CLTs and NK cells	Enhances the anti-virus properties of cytotoxicity, polyfunctionality, and multispecificity of HBV-specific T cells; combination treatment with IL-12 favors HBV clearence	Xiong et al. (2007); Wu et al. (2015); Zeng et al. (2013); Carreno et al. (2000)
IL-21	B cell differentiation, germinal center responce and antibodies production	Boosts and sustains HBV-specific CD8^+^ T cell effects by enhancing both cytolytic and noncytolytic pathways; associated with age-dependent outcome and vertical transmission of HBV infection	Li et al. (2015); Shen et al. (2021) Publicover et al. (2011); Vyas et al. (2019); Wang et al. (2021)
IL-17	Proinflammatory	Suppresses HBV replication in a noncytopathic manner; involved in the pathogenesis of liver damage, LC and HCC	Wang et al. (2013a); Bao et al. (2017)
IL-22	Tissue regeneration	Exerts dual effects in the context of inflammation	McAleer and Kolls. (2014)
IL-23	Stimulation of DC antigen presentation, generation, and maintenance of Th17 cells	Amplifies Th17 cell responses and liver inflammation; alters macrophage function and shapes a pro-cancer milieu for HCC	McKenzie et al. (2006); Wang et al. (2013b); Zang et al. (2018)
IFN	Control viral replication and dissemination	IFN-α exerts both direct antiviral and host immunomodulation effects and is the current standard treatment of HBV	Biron (2001); Sadler and Williams (2008)
		HBV specific IFN-γ producing T cells are associated with viral clearance	Wang et al. (2020)
TNF-α	Proinflammatory	Mediator of anti-HBV immunity, induces liver inflammation, leads to liver fibrosis	Wang et al. (2020)
IL-10	Regulatory cytokine, anti-inflammatory	Circulating IL-10+ Bregs and Tfr cells are associated with poor virus eradication and liver injury in CHB; IL-10-producing Breg cells suppress HBV-specific CD4^+^ and CD8^+^ T cell responses but enhance Treg cells	Wang et al. (2014); Das et al. (2012)
IL-35	Exerts an immunosuppressive effect on T cells	Elevates viral-specific Tregs, IL-10 production, decreases IL-17 secretion and contributes to viral persistence	Yang et al. (2019)
TGF-β	Anti-inflammatory cytokine, regulates diverse cellular processes	Boosting the activities of Treg cells; contributes to all stages of liver disease progression	Karimi-Googheri et al. (2014); Dooley and ten Dijke, (2012)
Chemokines			
CXCL9, CXCL10, CXCL11	Ligands of CXCR3, key immune chemoattractants during IFN-induced inflammatory response	Serum CXCL9, CXCL10, CXCL11 are elevated in CHB patients; CXCL10 enhances the migration of peripheral leukocytes into the liver; useful predictive indicators of disease progress and treatment response	Kakimi et al. (2001); Keating et al. (2014); Zhou et al. (2010); Sonneveld et al. (2013); Guo et al. (2016); Lee et al. (2013a)
CXCL13	Ligands of CXCR5, involed in the onset and maintenance of humoral immunity	Favors the recruitment of CD19^+^ B cells and CXCR5+CD8^+^ T cells into the liver; plasma CXCL13 serve as a biomarker for GC activity; increased plasma CXCL13 is distinctly observed in patients who achieve immune control of CHB infection	Li et al. (2020), (Havenar-Daughton et al., 2016), (Liu et al., 2017b)
CXCL8	Proinflammatory signaling cascade and guides neutrophils to infection site	Associated with the severity of liver inflammation/fibrosis and resistance to IFN-α therapy	Yang et al. (2014)
CXCL12	Strong chemotaxis for lymphocytes	Involved in recruitment and retention of immune cells in CHB patients with advanced liver fibrosis	Wald et al. (2004)

HBV, hepatitis B virus; IL, interleukin; IFN, interferon; NK, natural killer; Treg, T regulatory; Th, T helper; HCC, hepatocellular carcinoma; CHB, chronic hepatitis B; LC, liver cirrhosis; CTLs, cytotoxic T lymphocytes; DC, dendritic cells; TNF, tumor necrosis factor; Bregs, B regulatory cells; Tfr, T follicular regulatory; TGF, transforming growth factor.
